# The complete mitochondrial genome of *Liara* (*Liara*) *longispina* (Orthoptera: Tettigonidae: Conocephalinae)

**DOI:** 10.1128/mra.00078-26

**Published:** 2026-09-24

**Authors:** Heming Wang, Guoqing Li, Xun Bian, Bin Zhang

**Affiliations:** 1 College of Life Sciences & Technology, Inner Mongolia Normal University 71203, Hohhot, China; 2 Key Laboratory of Ecology of Rare and Endangered Species and Environmental Protection, (Guangxi Normal University), Ministry of Education 12388, Guilin, China; 3 Key Laboratory of Biodiversity conservation and Sustainable utilization for College and University of Inner Mongolia Autonomous Region, Hohhot, China; University of California Riverside, Riverside, California, USA

**Keywords:** mitochondrial genome, Tettigoniidae Conocephalinae, *Liara (Liara) longispina*

## Abstract

The complete mitochondrial genome (mitogenome) of *Liara* (*Liara*) *longispina* Zhang, Lu, & Bian (2022) was sequenced and annotated from Guangxi, China. The circular double-stranded mitogenome has a size of 16,402 bp and contains 13 protein-coding genes (PCGs), 22 transfer RNA genes (tRNAs), 2 ribosomal RNA genes (rRNAs), and a control region (CR/D-loop).

## ANNOUNCEMENT

The genus *Liara* belongs to the tribe Agraeciini, subfamily Conocephalinae, and family Tettigoniidae within the order Orthoptera (1). Its distribution spans Indo-China and Malesia and extends to regions including the Philippines, with recent records confirming its presence in Guangxi, China (2, 3). At present, the research of *Liara* is mainly based on morphological description (such as the identification of male genitalia) (3, 4), and to our knowledge, there is no report on the mitochondrial genome of this species. This study intends to annotate the mitochondrial genome sequencing of *Liara* (*Liara*) *longispina* species to fill the gap in molecular data for the genus (5).

The specimen of *Liara* (*Liara*) *longispina* was collected on August 18, 2023, from Diding Nature Reserve in Jingxi, Guangxi (23.112°N, 105.971°E). The specimen was immediately fixed in 100% ethanol and stored at −4°C. The voucher specimen was deposited in Guangxi Normal University (Guangxi, China) under the care of Xun Bian (xunbian2010@163.com).

Genomic DNA was extracted from hind leg muscle tissue dissected from the specimen using the TIANamp Genomic DNA Kit (TIANGEN, Beijing, China). The 150 bp paired-end library was constructed with the MGIEasy Kit (MGI) and sequenced on an Illumina NovaSeq 6000 (Illumina Inc.) (6). The Illumina NovaSeq 6000 generated 25.67 million raw reads (7.7 Gb), matching the SRA entry SRR36705941. Raw sequencing data were filtered using fastp v.0.20.0 to remove low-quality reads by trimming adapters and primers, and discarding reads with a Phred quality score <Q5 or N base number >3 (7). The filtered clean reads were used for mitogenome assembly with NOVOPlasty v.4.3.3 (parameters: assembly type = mito, genome range = 14,000–18,000 bp, k-mer = 39), using *Phlugiolopsis tuberculata* (NC_068779) as the reference sequence (8). The assembled mitogenome was annotated using the MITOS2 online platform (9). The mitochondrial genome map was mapped using Chloroplot 0.2.4 (10).

**Fig 1:**
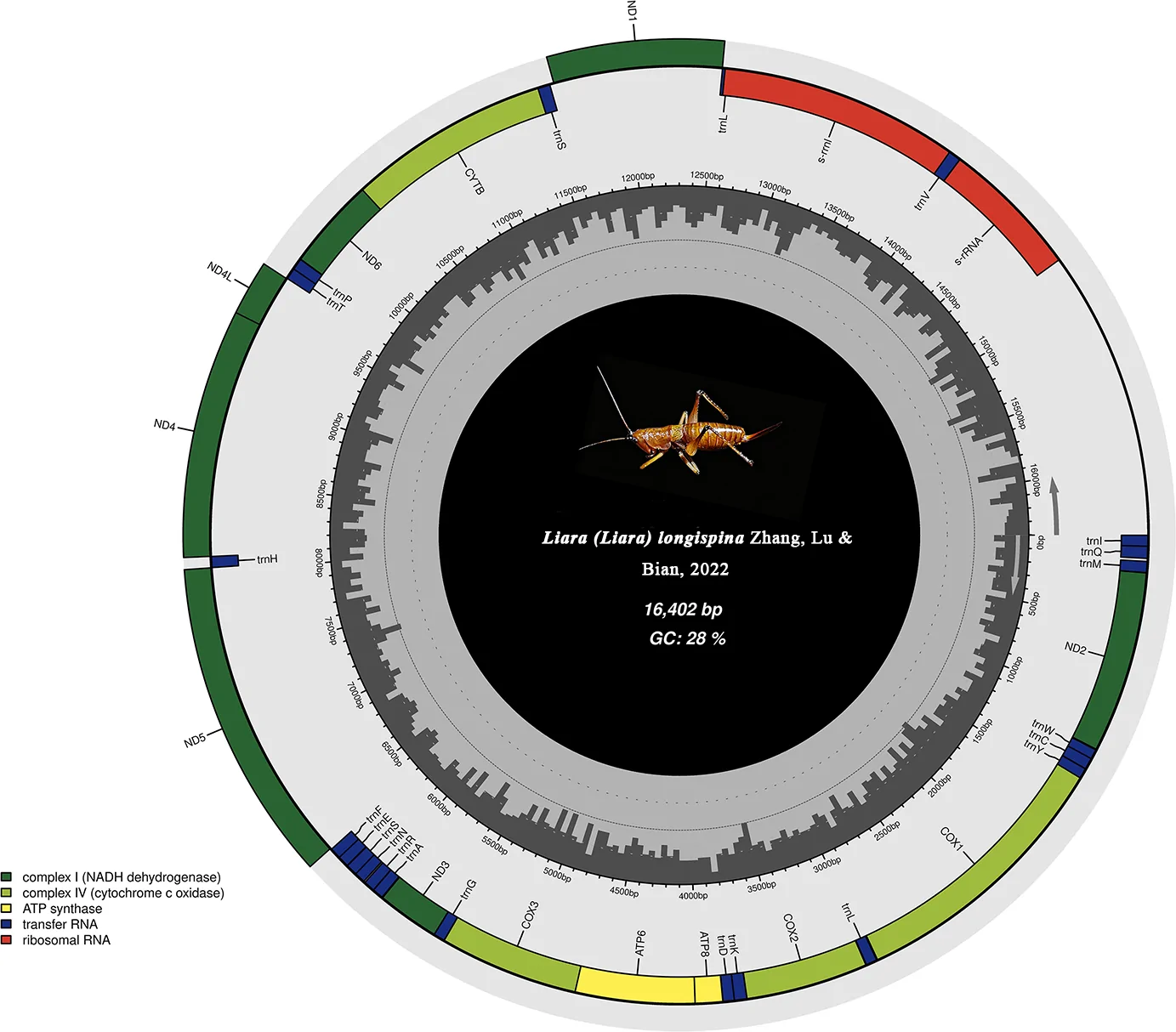
The circular map of the complete mitogenome of *Liara* (*Liara*) *longispina.*

The complete mitogenome of *Liara* (*Liara*) *longispina* (GenBank accession number PX795013) is 16,402 bp in length, with a typical circular double-stranded structure of metazoan mitochondria. It includes 37 genes: 13 PCGs, 22 tRNAs, 2 rRNAs, and 1 control region (CR) (Fig. 1). The mitogenome is AT-rich, with an A + T content of 72%; GC content is 28% (Table 1). Among the 13 PCGs, 9 PCGs (*nad2*, *cox1*, *cox2*, *atp8*, *atp6*, *cox3*, *nad3*, *nad6*, and *cob*) were on the forward strand, and 4 PCGs (*nad5*, *nad4*, *nad1*, and *nad4L*) were on the reverse strand (Fig. 1). The longest PCG is *nad5* (1,735 bp), and the shortest is *atp8* (162 bp). The 12S rRNA is 871 bp in length, located between *trnV* and *CR*, and the 16S rRNA is 1,301 bp in length, located between *trnL* and *trnV*; both are encoded on the reverse strand. The *CR* is 1,557 bp in length, located between 12S rRNA and *trnI*.

**TABLE 1:** Gene composition and annotation of *Liara* (*Liara*) *longispina*^*a*^

Gene	Location	Length (bp)	Type	Anti-codon	Direction	H/L Strand
*tRNA-Ile*	1–64	64	tRNA	GAT	Forward	+
*tRNA-Gln*	62–130	69	tRNA	TTG	Reverse	-
*tRNA-Met*	147–210	64	tRNA	CAT	Forward	+
*nad2*	211–1,239	1,029	CDS		Forward	+
*tRNA-Trp*	1,243–1,308	66	tRNA	TCA	Forward	+
*tRNA-Cys*	1,301–1,363	63	tRNA	GCA	Reverse	-
*tRNA-Tyr*	1,364–1,429	66	tRNA	GTA	Reverse	-
*cox1*	1,425–2,963	1,539	CDS		Forward	+
*tRNA-Leu*	2,966–3,029	64	tRNA	TAA	Forward	+
*cox2*	3,032–3,722	691	CDS		Forward	+
*tRNA-Lys*	3,723–3,793	71	tRNA	CTT	Forward	+
*tRNA-Asp*	3,793–3,857	65	tRNA	GTC	Forward	+
*atp8*	3,858–4,019	162	CDS		Forward	+
*atp6*	4,013–4,687	675	CDS		Forward	+
*cox3*	4,687–5,475	789	CDS		Forward	+
*tRNA-Gly*	5,474–5,537	64	tRNA	TCC	Forward	+
*nad3*	5,538–5,891	354	CDS		Forward	+
*tRNA-Ala*	5,895–5,958	64	tRNA	TGC	Forward	+
*tRNA-Arg*	5,958–6,021	64	tRNA	TCG	Forward	+
*tRNA-Asn*	6,030–6,095	66	tRNA	GTT	Forward	+
*tRNA-Ser*	6,091–6,156	66	tRNA	GCT	Forward	+
*tRNA-Glu*	6,158–6,222	65	tRNA	TTC	Forward	+
*tRNA-Phe*	6,221–6,284	64	tRNA	GAA	Reverse	-
*nad5*	6,285–8,019	1,735	CDS		Reverse	-
*tRNA-His*	8,020–8,082	63	tRNA	GTG	Reverse	-
*nad4*	8,083–9,421	1,339	CDS		Reverse	-
*nad4l*	9,415–9,711	297	CDS		Reverse	-
*tRNA-Thr*	9,714–9,778	65	tRNA	TGT	Forward	+
*tRNA-Pro*	9,778–9,843	66	tRNA	TGG	Reverse	-
*nad6*	9,845–10,366	522	CDS		Forward	+
*cob*	10,366–11,502	1,137	CDS		Forward	+
*tRNA-Ser*	11,501–11,570	70	tRNA	TGA	Forward	+
*nad1*	11,594–12,541	948	CDS		Reverse	-
*tRNA-Leu*	12,542–12,604	63	tRNA	TAG	Reverse	-
*16sRNA*	12,608–13,908	1,301	rRNA		Reverse	-
*tRNA-Val*	13,905–13,975	71	tRNA	TAC	Reverse	-
*12sRNA*	13,975–14,845	871	rRNA		Reverse	-
*CR*	14,846–16,402	1,557	D-LOOP		Reverse	-

^*a*^ –, light strand; +, heavy strand.

## Data Availability

The complete mitochondrial genome sequence of *Liara* (*Liara*) *longispina* is available in GenBank under accession number PX_795013. The associated BioProject, SRA, and BioSample numbers are PRJNA1399132, SRR36705941, and SAMN54473912, respectively. The mitochondrial genome referenced in the text is *Phlugiolopsis tuberculata* GenBank accession number NC_068779.

## References

[1] Cigliano MM, Braun H, Eades DC, Otte D. 2022. Liara (*Liara*) *longispina* Zhang, Lu & Bian, 2022. Orthoptera Species File. Available from: http://orthoptera.speciesfile.org/otus/842172/overview. Retrieved 28 Oct 2025.

[2] Zhang Q, Lu X, Bian X. 2022. One new species of *Liara* (Orthoptera: Tettigoniidae: Conocephalinae; Agraeciini) from Guangxi. Zootaxa 5200:595–600. doi:10.11646/zootaxa.5200.6.7 37045013

[3] Ingrisch S. 2020. New subgenera and species of *Agraeciini* (Orthoptera, Tettigoniidae, Conocephalinae) from South Asia found in historical insect collections. EvolSyst 4:119–132. doi:10.3897/evolsyst.4.60525

[4] Bian X. 2022. Description of the female of Liara (*Liara*) *guangxiensis* Bian, Zhang & Li, 2022 (Orthoptera: Tettigoniidae: Conocephalinae: Agraeciini). Zootaxa 5369:297–300. doi:10.11646/zootaxa.5369.2.9 38220712

[5] Bian X, Zhang Q, Li S. 2022. First report of the genus *Liara* (Orthoptera: Tettigoniidae: Conocephalinae: Agraeciini) from Guangxi, China with description of two new species. Zootaxa 5195:171–178. doi:10.11646/zootaxa.5195.2.6 37045298

[6] Pang S, Bian X. 2024. One new species the subgenus *Liara* (*Liara*) (Orthoptera: Tettigoniidae: Conocephalinae: Agraeciini) from Yunnan, China. Zootaxa 5486:557–562. doi:10.11646/zootaxa.5486.4.5 39646820

[7] Chen S, Zhou Y, Chen Y, Gu J. 2018. Fastp: an ultra-fast all-in-one FASTQ preprocessor. Bioinformatics 34:i884–i890. doi:10.1093/bioinformatics/bty560 PMC6129281 30423086

[8] Dierckxsens N, Mardulyn P, Smits G. 2017. NOVOPlasty: *de novo* assembly of organelle genomes from whole genome data. Nucleic Acids Res 45:e18. doi:10.1093/nar/gkw955 PMC5389512 28204566

[9] Luo T, Luo H, Guo J, Bian X. 2025. Complete mitochondrial genome of *Pseudocentema liui* (Phasmatodea; Lonchodidae). Microbiol Resour Announc 14:e00622-25. doi:10.1128/mra.00622-25 PMC12424333 40788024

[10] Bernt M, Donath A, Jühling F, Externbrink F, Florentz C, Fritzsch G, Pütz J, Middendorf M, Stadler PF. 2013. MITOS: improved de novo metazoan mitochondrial genome annotation. Mol Phylogenet Evol 69:313–319. doi:10.1016/j.ympev.2012.08.023 22982435

